# Principal Component Analysis of Munich Functional Developmental Diagnosis

**DOI:** 10.3390/pediatric13020031

**Published:** 2021-05-02

**Authors:** Grażyna Pazera, Marta Młodawska, Jakub Młodawski, Kamila Klimowska

**Affiliations:** 1 Collegium Medicum, Jan Kochanowski University in Kielce, 25-369 Kielce, Poland; g.pazera@op.pl (G.P.); jakub.mlodawski@ujk.edu.pl (J.M.); 2 Clinic of Neonatology, Provincial Combined Hospital in Kielce, 25-736 Kielce, Poland; 3 Department of Obstetrics and Gynaecology, Provincial Combined Hospital in Kielce, 25-736 Kielce, Poland; 4 Esculap Student’s Scientific Society, Jan Kochanowski University in Kielce, 25-369 Kielce, Poland; kklimo1997@gmail.com

**Keywords:** Munich functional developmental diagnosis, principal component analysis, neurodevelopment, psychomotor impairment

## Abstract

Objectives: Munich Functional Developmental Diagnosis (MFDD) is a scale for assessing the psychomotor development of children in the first months or years of life. The tool is based on standardized tables of physical development and is used to detect developmental deficits. It consists of eight axes on which the following skills are assessed: crawling, sitting, walking, grasping, perception, speaking, speech understanding, social skills. Methods: The study included 110 children in the first year of life examined with the MFDD by the same physician. The score obtained on a given axis was coded as a negative value (defined in months) below the child’s age-specific developmental level. Next, we examined the dimensionality of the scale and the intercorrelation of its axes using polychoric correlation and principal component analysis. Results: Correlation matrix analysis showed high correlation of MFDD axes 1–4, and MFDD 6–8. The PCA identified three principal components consisting of children’s development in the areas of large and small motor skills (axis 1–4), perception (axis 5), active speech, passive speech and social skills (axis 6–8). The three dimensions obtained together account for 80.27% of the total variance. Conclusions: MFDD is a three-dimensional scale that includes motor development, perception, and social skills and speech. There is potential space for reduction in the number of variables in the scale.

## 1. Introduction

Munich Functional Developmental Diagnosis (MFDD) is one of the methods that allows one to assess the psychomotor development of children in the first months or years of life. Due to its simplicity, the scale is able to diagnose a disorder of a particular developmental function, thanks to which we can implement precise medical interventions. Despite its simplicity and ease of use, the scale is not widely known. MFDD is a tool based on standardized tables of physical development according to Hellbrugge and Pechstein [1] and is primarily used to detect developmental delays or deficits.

This early diagnostic system was developed on the basis of observations of healthy infants and toddlers and on the basis of experience gathered during examinations of thousands of infants affected in various ways by disorders, developmental delays and disabilities. This information allowed the establishment of methodical and pediatric “standards” [2].

MFDD provides an early diagnosis of the eight most important psychomotor functions in infancy, i.e., crawling (1), sitting (2), walking (3), grasping (4), perception (5), speaking (6), speech understanding (7), social skills (8) [3]. This diagnostic system is based on the observation of children’s behavioral patterns, which change with the development of particular functions as a result of children’s mastery of skills during specific months of life. In MFDD, each month of life is characterized by those modes of behavior that were implemented by 90% of the children analyzed at that age. Thus, the method is based on the concept of “minimal behavior”, i.e., behavior that was demonstrated by 90% of the examined children in the corresponding month of life, and not on the average value or average behavior. In this way, a classification of typical behavioral patterns to a specific calendar age of the child was obtained [2]. This study aimed to validate and assess the dimensionality of this scale using statistical methods based on clinical data obtained after examining 110 children with the MFDD during their first year of life.

Principal component analysis (PCA) was used for this purpose. PCA is a statistical method whose main purpose is to reduce the number of quantitative variables in cases of multiparameter databases. This method also has other advantages. It allows us to visualize the structure of data in a multidimensional graphical view and the relationships and connections between individual variables. In mathematical terms, PCA allows us to extract new, mutually uncorrelated main factors from the variables we have. These factors define orthogonal spaces in which we can project the held variables. PCA can be used as a geometric representation of the formal structure of a questionnaire or test. In the context of the above information, the concept of factor load is also extremely important because it explains the correlation of individual clinical variables with the extracted principal components, thus describing the contribution of the variable to the individual newly extracted components. The cross-correlation of the MFDD axes allows us to extract the main domains of the research questionnaire on the basis of the PCA.

## 2. Materials and Methods

The study included 110 children (65 boys and 45 girls) and was part of a larger, yet unpublished investigation evaluating child development at 12 months of age using MFDD. The sample size was selected on the basis of the available literature [4]. Observations/variables ratio was 13:75. We evaluated full term and preterm infants ≥32 weeks of pregnancy, born from January 2015 to December 2018. In the case of preterm newborns, we used the corrected age, according to the assumptions of the method. We excluded from the study newborns with birth defects, central nervous system (CNS) bleeding, intrauterine hypoxia, intrauterine hypotrophy, gastrointestinal diseases, i.e., NEC and unaccompanied children (without parental care). Infants were examined during a special meeting in response to an invitation from our clinic. The examination was performed in the Department of Neonatology providing a standardized examination setting, such as a properly prepared room, the presence of only one related adult accompanying the child, examination of the child in optimal conditions, i.e., full, well-rested, not in the course of infection. All children were examined by the same pediatric neurologist. Parents signed an informed consent for the examination of the child, the model of which was previously presented to the bioethics committee.

The study was approved by the bioethics committee at Jan Kochanowski University in Kielce (no. 14/2016). The analysis was performed using Statistica 13.1 software (Tibco Software Inc., Palo Alto, CA, USA).

The MFDD uses ordinal assessment in number of months child is delayed behind average skills to actual age. It consists of assessing whether the given task was solved or not. There is no grading of the assessment. A developmental assessment sheet is used to record the results of each task.

The manner of behavior is assigned to the corresponding concrete month, and the age grades are separated by 1 month.

In the case of a child’s failure to achieve typical behavior patterns in a particular month of age, the data were coded as total negative values corresponding to the number of months by which the child shows a delay in the development of a particular function [2].

## 3. Results

Kaiser-Meyer-Olkin measure of sampling adequacy was 0.72 for dataset, Bartlett’s test of sphericity

The result (*p* < 0.005) showed that the correlation between specific questions in questionnaire was sufficiently large for PCA. The polychoric correlation matrix of the individual variables is shown in Table 1.

The matrix shows that the MFDD axes 1–4 are significantly correlated; the correlation was very high (0.802–0.922). Very high correlation strength was also obtained between MFDD axes 6, 7 and 8 (0.78–0.99).

**Figure 1:**
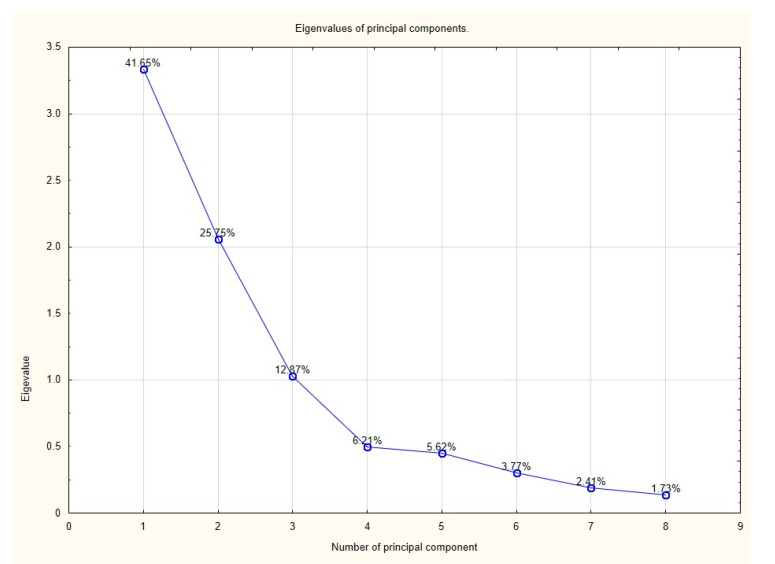
Scree plot. Each point is described by the percentage of the total variance explained by that component.

**Figure 2:**
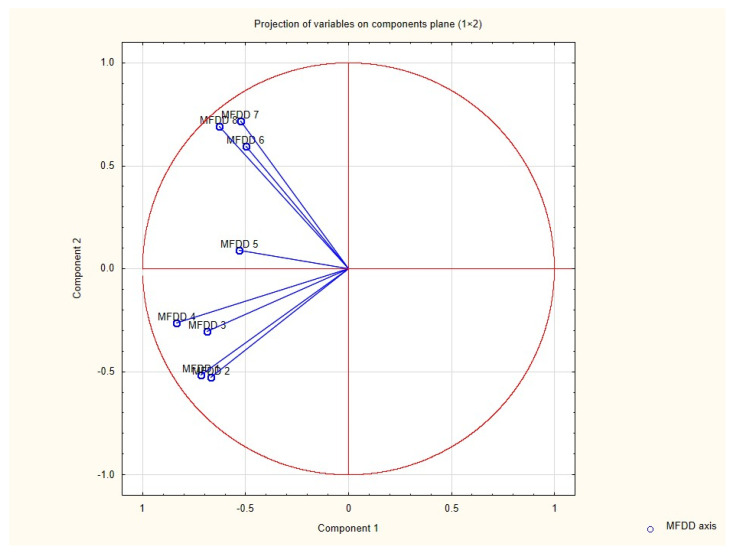
Loading plot. Plane defined by the first two components. Axes described by correlation value (factor load of the variable).

Results of the PCA showed that three components had eigenvalues greater than 1; based on Kaiser’s criterion we rejected the rest of the components. Principal components along with the percentage of explained variance are presented in a screen plot (Figure 1) and Table 2. Factor loads of individual variables are presented in Table 3. Loading plot (Figure 2—correlation circle) shows how strongly each variable influences the first two components. The plot is projected onto the plane defined by the first two principal components.

The confirmatory factor analysis (CFA) using weighted least squares (WLS) evidenced validity for three-factor model. We examined fit using Steiger-Lind root-mean-square error of approximation (RMSEA) and chi-square. Fit statistics for the scale were chi^2^ (d = 18) = 66, *p* < 0.001, RMSEA = 0.078. The data obtained indicate reasonable approximate fit of the model. We obtained the following reliability of subscales α_I_ = 0.76, α_III_ = 0.756, subscale II has only one item (axis 5).

## 4. Discussion

The development of a child since infancy is multifaceted and involves many aspects, which makes a global assessment of development of limited use. A global developmental quotient would obliterate the differentiation of diagnostic perceptions and thus could not be an indicator for therapeutic needs of a child. Distinguishing differentiated areas encompassing the eight functions mentioned above does not permit assessment of development as a whole, but it does allow recognition of a delay in each of the functions examined, which is sufficient for practical purposes. From the therapeutic point of view, the aim is not to identify children developing normally or above average, but to select those infants who require appropriate therapy due to developmental delay. Identification of the problem and early implementation of appropriate treatment gives a chance to help disabled children.

Although in the MFDD the individual areas of development are analyzed separately, in each case there is a need to take into account the relationships between the different types of functions. For this purpose, we use a developmental profile of the child, which we plot on a special sheet after establishing values for individual areas of function. The developmental profile is analyzed in terms of negative deviations, i.e., any deviation downwards from the calendar age or corrected age in the case of preterm infants should draw the attention of the examiner. A delay of two months in a particular function in the first year of life always creates a suspicion of pathology [5] and requires further explanation, whereas a deviation of one month certainly requires control but does not have to indicate a pathology.

The developmental profile should be analyzed as a whole, and it should be verified whether the individual functions affected by the delay are interrelated, e.g., whether all motor functions are delayed, whether the main area of delay lies in mental or social development, etc. If the delay affects all functions uniformly and the course of the profile ranks uniformly low in relation to age, with no clear deviations, we speak of a general developmental delay, which often occurs in children with severe mental disorders [2].

MFDD is a quick, easy-to-apply, and useful method for assessing motor skills in children born prematurely. Ortiz-Calderón et al. demonstrated high correlation between corrected age and motor age according to the MFDD method [6]. The same group of researchers also demonstrated that the MFDD is a useful tool for assessing language skill development in preterm infants [7]. MFDD is also used to assess the progress of children with developmental disabilities, which in the future may help to create certain developmental patterns in children with specific developmental disorders [8]. Markiewicz et al. demonstrated that developmental disorders, such as autism, intellectual disability, and specific language impairment, have a specific MFDD profile in respect to measures of cognitive function, social skills, and verbal communication [9].

PCA revealed the existence of three dimensions uncorrelated with each other in the diagnostic tool under study. The three principal components determined during PCA account for 80.27% of the total variance. The first component explaining 41.6% of the total variance correlates most strongly with axes 1–4 which correspond to the child’s motor skills. The second component accounting for 25.7% of the total population variance correlates most strongly with variables 6–8 which correspond to speaking skills, speech comprehension and social skills. The third component correlates most strongly with MFDD axis 5, which examines the child’s perceptual skills. Theoretically, the diagnostic tool can be simplified to three axes losing only 20% of the tested variance. We tested the obtained model with confirmatory factor analysis (CFA), obtaining a reasonable approximate fit. However, this result should be interpreted with caution because, in the case of CFA, the sample size should be close to 200 [10]. The model should therefore be tested on a larger population. Considering the inter-relationships of some developmental functions represented by specific axes in the MFDD scale [8], such “narrowed” assessment could inappropriately affect therapeutic strategies, and therefore it requires further study with re-validation.

The greatest variance in the study population results from the first four axes. The first three motor functions, i.e., crawling, sitting and walking, provide a measure of the development of the so-called “large motor”. The fourth axis, grasping, determines fine motor skills of the hand.

According to the PCA method, perception is a separate dimension that develops independently of the child’s motor, language and social skills, which may result from the complexity of the perception process. Diagnosis of “age of perception” in MFDD is carried out by means of special objects, which are standardized test materials. Each month of a child’s life is assigned a task to be performed with a particular object. It is primarily an orienting test for the senses of hearing and sight, as well as for the degree of concentration of attention on the environment and the understanding of interdependencies within it. Perception is the perceiving with the senses and further processing of stimuli in the central structures. With the development of perception, the child improves the ability to distinguish the quality and intensity of stimuli, as well as to select an individual stimulus from the background and to associate it with other perceptions and past experiences. A delay in perceptual development in infancy does not yet provide a basis for an unequivocal conclusion of mental retardation, and the reasons for such a delay may be varied (e.g., sensory disorders, motor impairment or deprivation) [2].

## 5. Conclusions

The MFDD is a three-dimensional scale that assesses children’s development in the areas of large and small motor skills (axis 1–4), perception (axis 5), active speech, passive speech and social skills (axis 6–8).

## Figures and Tables

**Table 1:** Polychoric correlation matrix of individual MFDD axes.

	MFDD 1	MFDD 2	MFDD 3	MFDD 4	MFDD 5	MFDD 6	MFDD 7	MFDD 8
MFDD 1	1.000	0.868	0.834	0.821	0.435	0.110	0.124	0.308
MFDD 2	0.868	1.000	0.835	0.922	0.523	0.061	0.263	0.424
MFDD 3	0.834	0.835	1.000	0.802	0.381	0.501	0.357	0.317
MFDD 4	0.821	0.922	0.802	1.000	0.804	0.407	0.443	0.666
MFDD 5	0.435	0.523	0.381	0.804	1.000	0.149	0.613	0.694
MFDD 6	0.11	0.061	0.501	0.407	0.149	1.000	0.780	0.844
MFDD 7	0.124	0.263	0.357	0.443	0.613	0.780	1.000	0.999
MFDD 8	0.308	0.424	0.317	0.666	0.694	0.844	0.999	1.000

**Table 2:** Originally determined principal components with matrix eigenvalues and percentage of variance explained.

Number of Component	Eigenvalue	% of Total Variance Explanation	Cumulative Variance [%]
1	3.332	41.649	41.649
2	2.060	25.752	67.401
3	1.030	12.870	80.270
4	0.497	6.206	86.476
5	0.450	5.617	92.093
6	0.301	3.767	95.860
7	0.192	2.406	98.265
8	0.139	1.735	100.000

**Table 3:** Factor loads of individual variables. Loads with correlation with component greater than 0.6 are marked with *.

Axis	Factor 1	Factor 2	Factor 3
MFDD 1	−0.717 *	−0.516	−0.020
MFDD 2	−0.669 *	−0.528	−0.021
MFDD 3	−0.687 *	−0.306	−0.370
MFDD 4	−0.836 *	−0.265	0.038
MFDD 5	−0.530	0.089	0.778 *
MFDD 6	−0.500	0.694 *	−0.520
MFDD 7	−0.525	0.717 *	0.063
MFDD 8	−0.628	0.690 *	0.103

## Data Availability

The data that support the findings of this study are openly available in OSF Storage at: DOI 10.17605/OSF.IO/NRQGK.

## References

[1] Hellbrügge T.H., Pechstein J. (1968). Entwicklungsphysiologische Tabellen für Säuglingsalter. Fortschr. Med..

[2] Hellbrugge T., Lajosi F., Menara D., Schamberger R., Rautenstrauch T., Kołodziej K., Banaszek G. (2014). Monachijska Funkcjonalna Diagnostyka Rozwojowa.

[3] Młodawska M., Pazera G., Młodawski J. (2021). Development of a preterm baby–an overview of current knowledge. Med. Stud..

[4] Mundfrom D.J., Shaw D.G., Ke T.L. (2005). Minimum Sample Size Recommendations for Conducting Factor Analyses. Int. J. Test..

[5] Ertan A.K., Tanriverdi H.A., Stamm A., Jost W., Endrikat J., Schmidt W. (2012). Postnatal neuro-development of fetuses with absent end-diastolic flow in the umbilical artery and/or fetal descending. Arch. Gynecol. Obstet..

[6] Ortiz-Calderón M.V., Valencia-Valencia D., Páez-Pineda O.D. (2017). Longitudinal evaluation of functional neurodevelopmental diagnosis according to the Munich Method in preterm infants. Rev. Salud Publica.

[7] Páez-Pineda O.D., Valencia-Valencia D., Ortiz Calderón M.V. (2014). Evaluating language acquisition using the Early Language Milestone (ELM) and Munich developmental scales. Rev. Salud Publica.

[8] Antonini U., Soldini E.A., D’Apuzzo V., Brunner R., Ramelli G.P. (2013). Longitudinal neurodevelopmental evolution in children with severe non-progressive encephalopathy. Brain Dev..

[9] Markiewicz K., Pachalska M. (2007). Diagnosis of severe developmental disorders in children under three years of age. Med. Sci. Monit..

[10] Moshagen M., Musch J. (2014). Sample size requirements of the robust weighted least squares estimator methodology. Eur. J. Res. Methods Behav. Soc. Sci..

